# Evaluating blood‐based biomarkers for Alzheimer's disease diagnosis across ethnic groups: a systematic review

**DOI:** 10.1002/alz70856_097986

**Published:** 2025-12-24

**Authors:** Natalia Chemas, Rifah Anjum, Charles R Marshall, Claudia Cooper, Ashvini Keshavan

**Affiliations:** ^1^ Queen Mary University of London, London, United Kingdom; ^2^ Queen Mary University of London, London, Greater London, United Kingdom; ^3^ Dementia Research Centre, UCL Queen Square Institute of Neurology, University College London, London, United Kingdom

## Abstract

**Background:**

Alzheimer's disease (AD) manifests differently across racial and ethnic groups, influenced by genetic and environmental factors. Blood‐based biomarker testing has the potential to enable early detection of AD, facilitating timely intervention and effective management. However, biomarkers validated only in homogenous populations may not accurately reflect disease presence, progression, or response to treatment in diverse groups. In this review, we assess the performance of blood‐based biomarker concentrations and AD clinical outcomes within studies conducted across different ethnoracial populations.

**Method:**

A comprehensive search of original scientific studies was conducted across Ovid MEDLINE, Embase, Web of Science, Scopus, and PubMed for articles published from 2000. Studies are included if they report blood‐based biomarkers (i.e., Aβ, *p*‐tau, t‐tau, NfL and GFAP) and their associations with AD clinical outcomes ‐ including cognitive performance, diagnostic accuracy, and disease progression ‐ and an ethnic or racial breakdown of the population sample. Data extraction focussed on study design, population demographics, biomarkers assessed, diagnostic metrics and statistical methods. Heterogeneity was evaluated using I^2^ statistics, and subgroup analyses were performed based on ethnoracial diversity and biomarker types.

**Result:**

A total of 3472 titles were imported, of which 109 articles were eligible for full‐text screening. Overall, we found a paucity of studies evaluating blood‐based biomarkers for AD across ethnically diverse populations. Individual studies focusing on the diagnostic performance of peripheral biomarkers as well as their variability across ethnoracial groups suggested that there are likely to be important variations in both biomarker levels and diagnostic performance across ethnoracial categories. However, the heterogeneity in measures and outcomes across the small number of relevant studies meant that high quality pooled evidence to guide clinical practice could not yet be derived.

**Conclusion:**

Blood‐based biomarkers for AD are likely to vary in their absolute concentrations and diagnostic performance across ethnoracial groups. High quality studies with harmonised exposures and outcomes are required to provide evidence about how this issue should influence clinical implementation and interpretation of blood‐based biomarkers in diverse real‐world settings.

